# The Long-lasting Radioprotective Effect of Caffeic Acid in Mice Exposed to Total Body Irradiation by Modulating Reactive Oxygen Species Generation and Hematopoietic Stem Cell Senescence-Accompanied Long-term Residual Bone Marrow Injury

**DOI:** 10.14336/AD.2019.0208

**Published:** 2019-12-01

**Authors:** Hyun-Jaung Sim, Govinda Bhattarai, Joshua Lee, Jeong-Chae Lee, Sung-Ho Kook

**Affiliations:** ^1^Department of Bioactive Material Sciences, Chonbuk National University, Jeonju 54896, South Korea; ^2^Cluster for Craniofacial Development and Regeneration Research, Institute of Oral Bioscience, Chonbuk National University School of Dentistry, Jeonju 54896, South Korea; ^3^Department of Bionanosystem Engineering, Graduate School, Chonbuk National University, Jeonju 54896, South Korea

**Keywords:** bone marrow injury, hematopoietic progenitor cells, reactive oxygen species, total body

## Abstract

Total body irradiation (TBI) serves as an effectively curative therapy for cancer patients and adversely causes long-term residual bone marrow (BM) injury with premature senescence of hematopoietic stem cells (HSCs), which is mediated by increased production of reactive oxygen species (ROS). In the present study, we investigated how the exposure time of TBI in a mouse model affects HSCs and whether the treatment of caffeic acid (CA), a known dietary phenolic antioxidant, has a radioprotective effect. Single (S)-TBI at a sublethal dose (5 Gy) caused relatively higher induction of mitochondrial ROS and senescence-related factors in HSCs than those in hematopoietic progenitor cells (HPCs) and Lineage^-^Sca-1^+^c-Kit^+^ (LSK) cells, as well as reduced clonogenic formation and donor cell-derived reconstituting capacity. Repetitive double (D)-TBI (two months after the S-TBI at a dose of 5Gy) further weakened HSPC function via mitochondrial ROS accumulation and senescence-associated β-galactosidase (SA-β-gal) activity. Oral administration of CA (20 mg/kg) five times before and once immediately after TBI ameliorated ROS generation and TBI-induced HSC senescence and its radioprotective effect was long lasting in S-TBI mice but not in D-TBI mice. Further, supplementation of CA also induced apoptotic cell death of colon cancer cells. Collectively, these findings indicate that CA has a dual effect, ameliorating HSC senescence-accompanied long-term BM injury in S-TBI mice and stimulating apoptotic cell death of colon cancer cells.

Although moderate or high doses of TBI are administered to cancer patients for treatment, this treatment causes acute myelosuppression and long-term residual BM injury with senescence of HSCs [1,2]. BM damage caused by TBI is reported to initiate from the immediate generation of reactive oxygen species (ROS) in HSCs and these initially generated ROS remain in the HSCs, inducing HSC senescence and inhibiting self-renewal capacity, which mediates long-term BM injury [3-5]. The prevention of bone marrow injury mediated by ROS in moderate or high-doses of TBI-exposed cancer patients is clinically needed to remove the occurrence of secondary adverse events. In line with this, antioxidant drugs have been studied as a way of treating TBI-induced BM injury [6]. However, the antioxidant effects of metformin and resveratrol in preventing BM injury are mostly proved in mice that have received single TBI and bred only for a short period after treatment [7, 8]. Such experimental models do not reflect the situation of cancer patients who must undergo subsequent cycles of radiotherapy as the occasion demands and require follow-up observations, as accumulated radiotoxic stress results in hematopoietic diseases such as hypoplastic anemia, leukemia and lymphoma. Therefore, the application of antioxidants with TBI-requiring cancer patients is a proper supplement to limit long-term residual BM injury. For clinical prospective, long-term study using experimental animals exposed to repetitive irradiation has still obscure and need to be explored. In this study, we investigated whether CA, a hydroxycinnamic acid present in many fruits, vegetables, and beverages such as coffee, wine or tea [9-11], had antioxidant effects in HSCs from mice exposed to single or repetitive TBI and whether treatment lasted long enough to limit ROS-mediated HSC senescence and long-term BM injury after TBI exposure. Further, the application of radiotherapy with an anti-cancer drug as a curative agent for the treatment of metastatic colon cancer, the anti-cancer effect of CA in the colon cancer cell line CT26 was also evaluated.

## MATERIALS AND METHODS

### Animals and treatment

All experimental procedures were approved by the Animal Welfare Committee of Chonbuk National University. C57BL/6 female mice (6 weeks old) were purchased from Damul Science (Daejeon, Korea) and equilibrated for seven days before the experiment. During the experimental period, mice were housed at 22 ± 1°C and 55 ± 5% humidity on an auto-cycling 12 h light/dark cycle with free access to food and water. Mice were exposed to 5 Gy TBI with Ɣ-rays by regulating dosage time that was based on the radioactive half-life of Ɣ-rays on a rotating platform (Model 109-85 series- JL Shepherd & associates, San Fernando, CA, USA). Two months after the first TBI, mice were again exposed to the same dose. CA was obtained from Sigma-Aldrich Co. LLC (St. Louis, MI, USA) and dissolved in 99% ethanol and diluted with distilled water before use. A final volume of 200 μl CA (20 mg/kg body weight) was administered five times (once every three days) before and once immediately after TBI to mice via oral gavage (Supplementary Figure S1). The dose of CA for animal studies was determined on the basis of previous studies [12-14]. To measure hematopoietic cells in BM, tibias and femurs from mice were dissected at 2 months after TBI and flushed with phosphate-buffered saline (PBS). Red blood cells (RBCs) in the flushed BM cells were removed with RBC lysis buffer (Sigma-Aldrich).

### Cell culture

Mouse colon cancer (CT-26), human liver cancer (HepG2) and human breast cancer (MCF-7) cells lines were purchased from ATCC and cultured in RPMI 1640 and DMEM medium supplemented with 10% FBS and antibiotics (penicillin G and streptomycin) at 37^°^C in a humidified atmosphere of 5% CO_2_.

### Flow cytometry

Numbers of hematopoietic cells derived from BM were analyzed by multicolor flow cytometry (BD Aria, BD Bioscience). These cell populations were further analyzed with FlowJo software for phenotypical identification. Hematopoietic progenitor cells (HPCs, Lin^-^Sca-1^-^c-Kit^+^), Lin^-^Sca-1^+^c-Kit^+^ (LSK) cells, and hematopoietic stem cells (HSCs, CD150^+^CD48^-^LSK) from BM were characterized with the following lineage markers: PE-Cy7-conjugated anti-CD3, anti-CD4, anti-CD8, anti-CD45R, anti-CD11b, anti-Gr-1, and anti-TER-119 (BD Biosciences); FITC or PE-conjugated anti-Sca-1 (BD Biosciences); APC-conjugated anti-c-Kit (BD Biosciences); PerCP/Cy5.5-conjugated anti-CD150 (eBioscience); or APC-Cy7-conjugated anti-CD48 (BD Biosciences). SA-β-gal activity and mitochondrial superoxide anion levels were assessed using C_12_FDG (Molecular Probes) and MitoSox^TM^ Red (Invitrogen), respectively. Expression of p16^INK4a^ was determined with Alexa Flour 488-conjugated antibody (Santa Cruz Biotechnology) after fixation and permeabilization (BD Bioscience).

### Colony-forming cell assays

For colony-forming unit (CFU) assay, BM cells (2×10^4^ cells per dish) were cultured in 35-mm dishes with MethoCult^®^ methylcellulose-based medium (MethoCult^®^ GF M3434) and the number of colonies formed was counted using standard criteria on day 12. For pre-B assays, BM cells (2×10^5^ cells per dish) were divided into 35-mm dishes with MethoCult® methylcellulose-based medium (MethoCult^®^ M3630), followed by the colony counting on day 7.

### Competitive transplantation assays

For donor-derived repopulating potential assays, 5×10^5^ BM cells from control, S-TBI, and D-TBI mice (CD45.2) were mixed with an equal number of cells from competitor mice (CD45.1) and then transplanted into recipient mice exposed to lethal irradiation (10 Gy). B6 × SJL F1 hybrid mice (CD45.1/CD45.2 double-positive) were used as recipients to distinguish donor-derived cells from host-derived cells. CD150^+^CD48^-^LSK cells (1×10^3^) from BM of control or S-TBI+CA mice (CD45.2, 2 years old) were co-transplanted with cells from competitor mice (CD45.1, 5 months old) into conditioned recipient mice (CD45.1/2). Peripheral blood (PB) cells were collected from recipient mice 5 months after transplantation. The ratio of CD45.1/CD45.2 was estimated by flow cytometry using FITC- and PE-conjugated antibodies (BD Biosciences).

### Blood tests

PB samples collected from cardiac puncture of mice were saved in Vacutainer® (BD Biosciences, Franklin Lakes, NJ, USA) plastic tubes coated with K_2_EDTA and measured in the Department of Laboratory Medicine of Chonbuk National University Hospital. To analyze levels of white blood cells (WBCs), RBCs, and platelets in samples, an automated complete blood cell counter (Sysmex XE-2100; TOA Medical Electronics Co., Kobe, Japan) was used.

### Measurement of intracellular ROS

A stock solution of 2’, 7’-dichlorodihydrofluorescein-diacetate (DCFH-DA) (Calbiochem, Germany) was prepared in DMSO and stored at -20°C in the dark. When CT-26 cells reached 90% confluence on 60-mm culture dishes, the cells were treated with CA for 24 h and incubated with 10 µM DCFH-DA for 30 min. The fluorescence of DCF was recorded at 515 nm (FL-1H) using a FACS Calibur® system (Becton-Dickinson, San Jose, CA, USA) and 10,000 events were counted per sample.

### Western blot analysis

Protein lysates were prepared from CT-26 cells treated with CA. Protein extracts (20 µg per sample) were separated by sodium dodecyl sulfate-polyacrylamide gel electrophoresis on 15% gels and electroblotted onto polyvinylidene difluoride membranes. Blots were washed with a solution containing 10 mM Tris-HCl (pH 7.6), 150 mM NaCl, and 0.05% Tween-20, blocked with 5% skim milk for 1 h, and then incubated with primary antibodies such as cleaved caspase-3 (Cell Signaling Technology, Danvers, MA, USA) and β-Actin (sc-47778 Santa Cruz Biotechnology, Santa Cruz, CA, USA). After washing, the membranes were incubated with horseradish peroxidase-conjugated goat anti-rabbit IgG or goat anti-mouse IgG. Immunoreactive bands were visualized by enhanced chemiluminescence (ELPIS-Biotech, Taejeon, Republic of Korea) followed by exposure to X-ray film (Eastman Kodak, Rochester, NY, USA).

### Nuclear morphology assessment

CT-26 cells were sub-cultured for 24 h on cover slips in twenty-four-well culture plates. For the observation of cell morphological change, the cells were treated with CA for 24 h, washed with ice-cold PBS, and then fixed with 2% formaldehyde and 0.2% glutaraldehyde for 30 min. After washing with PBS, cell nuclei were stained with 4,6-Diamidino-2-phenylindole dihydrochloride (DAPI, 1.5 µg/mL) and nuclear morphology was visualized by fluorescence microscopy (Olympus JP/ iX71-21PH, Tokyo, Japan).

### Statistical analysis

All results were expressed as the mean ± standard deviation (SD). One-way analysis of variance was used for multiple comparisons using SPSS version 16.0 software (Chicago, IL, USA). Student’s *t*-test was used to determine significant differences; * < 0.05, ** < 0.01, *** < 0.001.

## RESULTS

### Repetitive TBI accumulates oxidative stress and promotes senescence in hematopoietic stem progenitor cells

**Figure 1.**
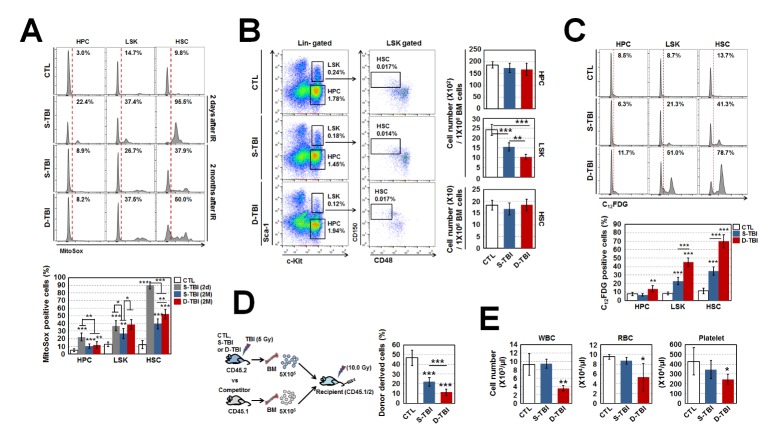
Repetitive TBI leads to the accumulation of oxidative stress and senescence of HSPCs. (A) Level of mitochondrial superoxide anions in HPCs, LSKs, and HSCs from BM of control (CTL), single total body irradiation (S-TBI), and double TBI (D-TBI) mice, assessed by multicolor flow cytometry using MitoSOX™ Red reagent at the indicated times (n = 8). (B) HSPCs from BM of control, S-TBI and D-TBI mice were phenotypically characterized and counted by flow cytometry (n = 8). Results were obtained from mice that were sacrificed 2 months after TBI exposure. (C) Senescence-associated β-galactosidase (SA-β-gal) activity was measured with C_12_FDG, a galactosidase substrate (n = 8). (D) For long-term competitive reconstituting potential assays, BM cells (5×10^5^) from control, S-TBI, and D-TBI mice (CD45.2) were co-transplanted with an equal number of BM cells from competitor mice (CD45.1/2) into lethally irradiated recipient mice (CD45.1/2, 10 Gy) (n = 7). (E) White blood cells (WBC), red blood cells (RBC) and platelets in the peripheral blood were measured by an automated complete blood cell counter (n = 7). All data are presented as mean ± SD. *p < 0.05, **p <0.01 and *** p < 0.001 vs. control, determined by Student’s *t*-test.

We compared mitochondrial ROS levels in hematopoietic stem progenitor cells (HSPCs) such as HPCs (Lin^-^Sca-1^-^c-kit^+^ cells), Lin^-^Sca-1^+^c-Kit^+^ cells (LSKs) and HSCs (CD150^+^CD48^-^LSK cells) [15] from mice exposed to single (S)- or double (D)-TBI at 5 Gy, as mentioned in experimental schedule (Supplementary Fig. S1). HSPCs from S-TBI mice sacrificed 2 days after TBI exhibited a considerable increase in mitochondrial ROS level compared with cells from control mice. In particular, HSCs (90.0%) exhibited a higher ROS level than HPCs (22.2%) and LSKs (36.8%) (Fig. 1A) [5]. Although the initial increase in ROS was reduced in S-TBI mice sacrificed at 2 months compared with 2 days after TBI, ROS levels in HPCs, LSKs and HSCs were still higher than in cells from control mice. D-TBI exposure caused greater ROS accumulation. Further, D-TBI mice sacrificed 2 months after TBI showed a significantly higher ROS level in LSKs and HSCs than cells from control and even S-TBI mice (Fig. 1A). In both S-TBI and D-TBI mice, HSCs retained relatively higher ROS levels compared to HPCs and LSK cells. These results indicate that TBI increases immediately and persistently the production of mitochondrial ROS in BM-conserved hematopoietic cells, of which HSCs are more vulnerable to TBI than HPCs and LSKs, and repetitive TBI leads to ROS accumulation.

We next determined how repetitive TBI modulated HSPC numbers. The number of LSKs, but not HPCs and HSCs, significantly decreased after S-TBI and decreased further in D-TBI mice compared with control mice (Fig. 1B). Since ROS are well known as key mediators in causing HSC senescence under ionizing irradiation, we measured SA-β-gal activity in hematopoietic cells [16]. Compared with S-TBI mice that had higher SA-β-gal activity in LSKs and HSCs than control mice, D-TBI mice exhibited significantly more intensified SA-β-gal activity (Fig. 1C). Expression of p16^Ink4a^, a molecule that is upregulated in senescent stem cells [17], was statistically increased in LSKs and HSCs from S-TBI and D-TBI mice compared with control mice. However, expression did not differ between S-TBI and D-TBI mice (Supplementary Fig. S2) [4]. CFU assays to evaluate HPC activity indicated that clonogenic formation was significantly reduced depending on the time of TBI exposure (Supplementary Fig. S3) [18]. To measure *in vivo* HSC activity, we performed competitive transplantation by co-transplanting an equal number of BM cells (5×10^5^) from control, S-TBI, or D-TBI mice (CD45.2) and cells from competitor mice (CD45.1) into lethally irradiated recipient mice (CD45.1/2, Fig. 1D). Compared with control BM cells, S-TBI BM cells had inferior reconstituting activity in the peripheral blood (PB) of recipients and D-TBI BM cells had less activity than S-TBI BM cells (Fig. 1D). The numbers of circulating WBCs, RBCs and platelets were found to be statistically reduced in D-TBI, but not S-TBI mice, when compared with control mice (Fig. 1E). Taken together, these findings suggest that repetitive TBI irreversibly results in the accumulation of radiotoxic stress, as well as senescence in HSPCs, leading to greater defects in their function.

### Oral administration of CA has the ability to completely prevent HSC senescence-accompanied long-term BM injury in S-TBI mice by modulating ROS generation

**Figure 2.**
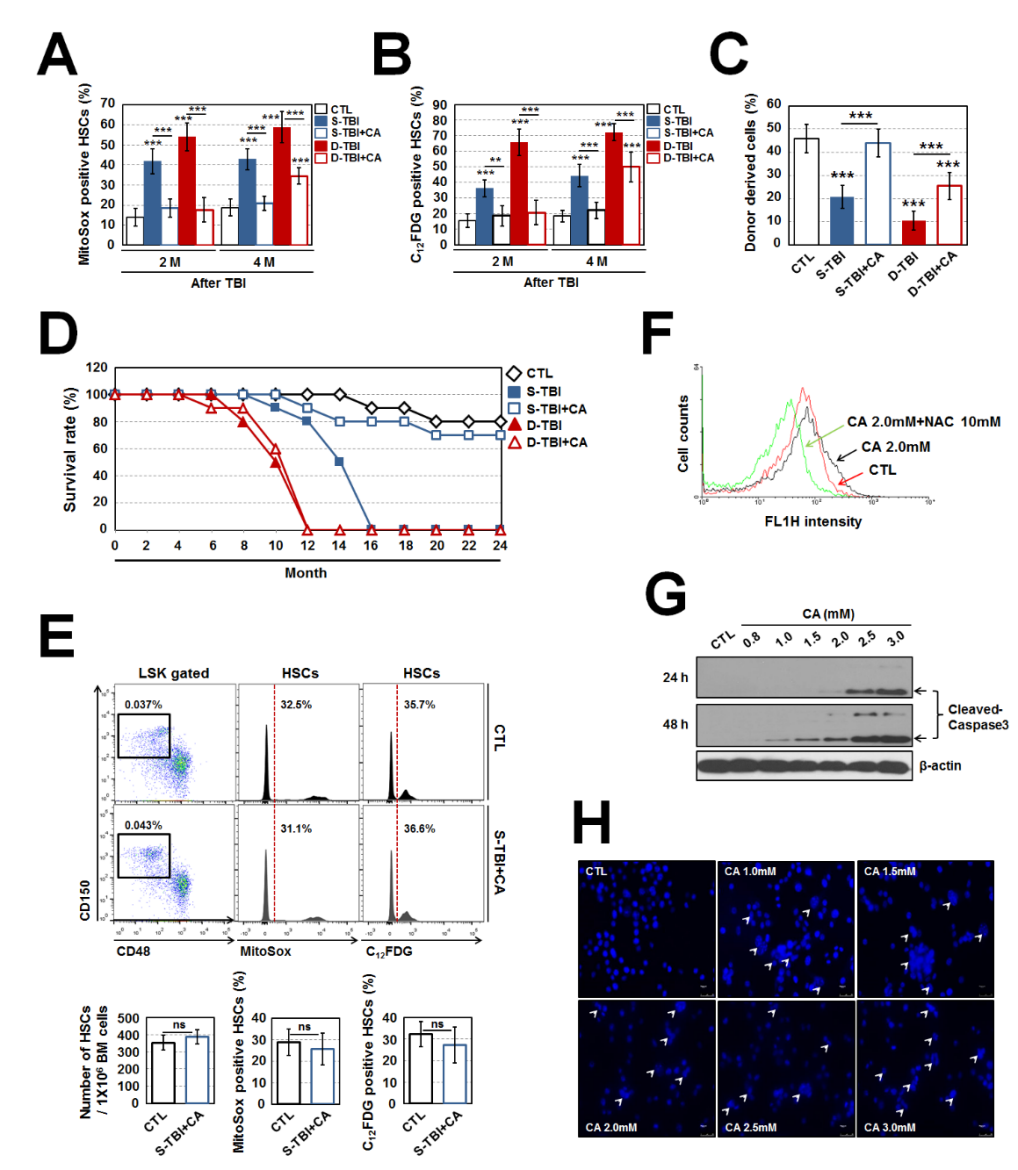
Supplementation of CA limits HSC senescence-accompanied long-term BM injury and improves survival in S-TBI mice by modulating ROS generation and induces apoptotic death of colon cancer cells. (A) Antioxidant effect of CA was evaluated in HSCs from BM of CA-treated mice exposed to TBI using MitoSOX™ Red reagent at the indicated times after TBI (n = 7). (B) Percentage of C_12_FDG-positive HSCs was determined by flow cytometry at the indicated times after TBI (n = 7). (C) For competitive transplantation experiments, equal numbers of CD150^+^CD48^-^LSK cells (1×10^3^) from control, S-TBI, CA-treated S-TBI, D-TBI, and CA-treated D-TBI (CD45.2) were co-transplanted with cells from competitor mice (CD45.1) into conditioned recipient mice (CD45.1/2, n = 7) that were lethally irradiated. Peripheral blood was collected from recipients at 5 months after transplantation and CD45.1/CD45.2 ratio was measured by flow cytometry. (D) Survival rate of mice exposed to TBI in combination with or without treatment of CA was measured (n = 10). (E) Number, mitochondrial ROS level and SA-β-gal activity of HSCs were assessed in controls and CA-treated S-TBI mice (more than 2 years old) (n = 6). (F) CT-26 colon cancer cells were treated with CA along with NAC for 24 h (a potent reactive oxygen species inhibitor) and the DCF fluorescence in the cells was determined by flow cytometry. Representative data are shown for three independent experiments. (G) CT-26 colon cancer cells were treated with the indicated concentrations of CA and the level of cleaved-caspase 3 was analyzed by western blotting 24 and 48 h after incubation. A representative result is shown from three independent experiments. (H) CT-26 colon cancer cells were stained with DAPI 24 h after CA treatment, in which arrow heads indicate apoptotic death with nuclear fragmentation. Representative data are shown for three independent experiments. All data are presented as mean ± SD. *p < 0.05, **p < 0.01 and *** p < 0.001 vs. control, by Student’s *t*-test and one-way analysis of variance using SPSS software (ver.12.0) for multiple comparisons.

To determine whether the suppression of TBI-mediated ROS limits the progress of HSC senescence, we administered CA, a known dietary phenolic antioxidant, to mice for the indicated periods (Supplementary Fig. S1) [19]. Not only CA-treated S-TBI mice, but also CA-treated D-TBI mice exhibited significantly ameliorated ROS levels and SA-β-gal activity in HSCs compared to corresponding non-treated mice at 2 months after TBI. Both the levels even recovered to the basal levels of control mice (Figs. 2A and B). However, the inhibitory potentials of supplemental CA on ROS accumulation and senescence induction in HSCs were diminished in D-TBI mice sacrificed 4 months after TBI. ROS levels and SA-β-gal activity began to significantly increase again in CA-treated D-TBI mice but not CA-treated S-TBI mice compared to control mice (Figs. 2A and B). To assess *in vivo* HSC activity in CA-treated TBI mice that were sacrificed 4 months after the exposure, we co-transplanted equal numbers (1×10^3^) of CD150^+^CD48^-^LSK cells from control, S-TBI, CA-treated S-TBI, D-TBI or CA-treated D-TBI mice (CD45.2) and competitor mice (CD45.1) into conditioned recipient mice (CD45.1/2). The low repopulating ability of S-TBI mice-derived HSCs in the recipient mice was undoubtedly improved by transplanting CA-treated S-TBI mice-derived HSCs up to the control mice-derived HSCs’ basal level (Fig. 2C). CA-treated D-TBI mice-derived HSCs also had enhanced reconstituting capacity compared with non-treated D-TBI mice-derived counterparts but did not show the similar potential to control mice-derived counterparts (Fig. 2C). Taken as a whole, our findings suggest that the treatment of supplemental CA is effective for limiting TBI-induced ROS generation and for maintaining HSC function by inhibiting HSC senescence, the effects of which are consistent or transient depending on the exposure time of TBI.

We subsequently investigated whether the oral administration with CA extends survival rate of mice exposed to sublethal TBI. Compared with control mice that showed 90% survival rate until 19 months, all mice in S-TBI and D-TBI groups died at 16 and 12 months, respectively, after TBI exposure (Fig. 2D). Oral administration of CA extended the survival rate of S-TBI mice, such that CA-treated S-TBI mice retained 70% survival even after 24 months post-TBI. However, the CA-mediated radioprotective effect on survival rate was not found in D-TBI mice (Fig. 2D). To determine whether the effect of supplementary CA consistently lasts over the lifespan, we sought to compare HSCs between 24-month old control and CA-treated S-TBI mice. As shown in Figure 2E, the number, ROS level and SA-β-gal activity of HSCs all were comparable between both the mice groups. No significant difference in donor cell-derived repopulating potential was found in the conditioned recipients (CD45.1/2) co-transplanted with an equal number of CD150^+^CD48^-^LSK cells (5×10^2^) from control or CA-treated S-TBI mice (CD45.2) and competitor mice (CD45.1) (Supplementary Fig. S4). Furthermore, CA-treated S-TBI mice maintained similar numbers of WBCs, RBCs and platelets in the PB compared to corresponding control mice (Supplementary Fig. S5). These findings suggest that the radioprotective effect of supplementary CA consistently lasts, granting normal HSC function and hematopoiesis, and thereby prolonging the survival of S-TBI mice.

### Supplementation of CA induces apoptotic death of colon cancer cells

Since a delicate balance of intracellular ROS levels is required for regulating cancer cell function [20], we further examined the underlying mechanisms of how a direct addition of CA affects ROS levels and viability of colon (CT-26), liver (HepG2) and breast (MCF-7) cancer cells. CA treatment (0-3 mM) decreased viability of the cells in a dose- and time-dependent manner (Supporting Information Fig. S6). CA treatment induced dose-dependent increases in the formation of cleaved-caspase 3 and nuclear fragmentation, indicating apoptotic cell death (Figs. 2F and G). Specifically, CA treatment (2 mM) moderately increased cellular ROS accumulation compared with the untreated control, whereas the CA-stimulated ROS generation was near completely inhibited in the cells treated together with 10 mM N-acetyl-L-cysteine (NAC) (Fig 2H). Overall, combined treatment with 2 mM CA and 10 mM NAC significantly restored the viability that had reduced by CA treatment alone similar to that of the untreated control cells (data not shown). These results indicate that CA exerts an anti-cancer effect on CT-26 cells by inducing apoptotic cell death, in which intracellular ROS levels are closely associated with the effect.

## DISCUSSION

Although TBI is an everlasting curative therapy, it causes long-term residual BM injury accompanied with ROS-associated HSC senescence [4]. In the clinic, a large number of cancer patients undergo cyclic radiotherapy by reason of extermination or relapse of a tumor and need a follow-up observation during their lifetime. As such, using and observing an animal model that is exposed to repetitive IR and treated with antioxidant drugs for preventing IR-mediated long-term BM injury is necessary to better understand cellular mechanisms for clinical cancer treatment. This current study highlights that repetitive TBI results in irreversible accumulation of radiotoxic stress in HSCs than single TBI. This study also demonstrates the antioxidant and radioprotective effect of supplementary CA by modulating HSC senescence-accompanied long-term BM injury and survival in S-TBI mice, but transiently in D-TBI mice.

It was found that the toxicity of CA was extremely low and the dose not lethal in animals [14]. Similarly, the radio-protective and anti-cancer effects of CA were already known [21-25]. However, the effect of CA on TBI-induced BM injury and the underlying mechanisms have not been studied. In this current study, we found that CA has a protective effect on HSC senescence-accompanied BM injury in TBI-exposed mice and has an anti-cancer effect in cancer cells. These findings indicate that supplementation of CA may improve the quality of life or minimize radiation-induced side effects in cancer patients. It seems that CA may be a clinically ideal candidate for use as an adjuvant in cancer therapies. Moreover, our *in vitro* studies showed that micromolar concentrations of CA did not induce apoptosis in cancer cells and required a higher concentration. Previous reports of anti-cancer effects of CA in other cancer cell lines used comparatively high concentrations of CA, consistent with our observations [ 23-25]. Of note, CA serves as a prooxidant in colon cancer cells and induces a caspase-dependent apoptosis, and it’s radiosensitizing effect in colon cancer cell already identified [26]. Although, TBI is rarely used for the treatment of colon cancer patients, it could be paired with CA as an alternative to chemotheraphy for the treatment of metastatic cancers originating from the colon. For example, colon cancer metastasizes to the liver, lungs [27] and bone, although bone metastases arising from colon cancer are rare, accounting for about 3-5% of bone metastases [28, 29].

Taken together, these findings indicate that CA could be clinically used as a supplemental drug to autonomously ameliorate HSC senescence-accompanied long-term BM injury and to stimulate apoptotic death of cancer cells in patients who need radiotherapy with chemotherapy. Further understanding of cell autonomous and non-autonomous mechanisms by which repetitive irradiation induces BM injury, as well as on the efficacy of dietary phenolic antioxidants in clinical applications for radiotherapy-required cancer patients will be needed.

## References

[1] Mauch P, Constine L, Greenberger J, Knospe W, Sullivan J, Liesveld JL, et al (1995). Hematopoietic stem cell compartment: acute and late effects of radiation therapy and chemotherapy. Int J Radiat Oncol Biol Phys, 31:1319-1339. 7713791 10.1016/0360-3016(94)00430-S

[2] Domen J, Gandy KL, Weissman IL (1998). Systemic Overexpression of BCL-2 in the hematopoietic system protects transgenic mice from the consequences of lethal irradiation. Blood, 91:2272-2282. 9516125

[3] Wang Y, Liu L, Pazhanisamy SK, Li H, Meng A, Zhou D (2010). Total body irradiation causes residual bone marrow injury by induction of persistent oxidative stress in murine hematopoietic stem cells. Free Radic Biol Med, 48:348-356. 19925862 10.1016/j.freeradbiomed.2009.11.005 PMC2818724

[4] Shao L, Feng W, Li H, Gardner D, Luo Y, Wang Y, Liu L, et al (2014). Total body irradiation causes long-term mouse BM injury via induction of HSC premature senescence in an Ink4a- and Arf-independent manner. Blood, 123:3105-3115. 24622326 10.1182/blood-2013-07-515619 PMC4023419

[5] Balaban RS, Nemoto S, Finkel T (2005). Mitochondria, Oxidants, and Aging. Cell, 120:483-495. 15734681 10.1016/j.cell.2005.02.001

[6] Xue XL, Han XD, Li Y, Chu XF, Miao WM, Zhang JL, Fan SJ (2017). Astaxanthin attenuates total body irradiation-induced hematopoietic system injury in mice via inhibition of oxidative stress and apoptosis. Stem Cell Res Ther, 8:7. 28115023 10.1186/s13287-016-0464-3 PMC5260077

[7] Xu G, Wu H, Zhang J, Li D, Wang Y, Wang Y, et al (2015). Metformin ameliorates ionizing irradiation-induced long-term hematopoietic stem cell injury in mice. Free Radic Bio Med, 87:15-25. 26086617 10.1016/j.freeradbiomed.2015.05.045 PMC4707049

[8] Zhang H, Zhai Z, Wang Y, Zhang J, Wu H, Wang Y, et al (2013). Resveratrol ameliorates ionizing irradiation-induced long-term hematopoietic stem cell injury in mice. Free Radic Biol Med, 54:40-50. 23124026 10.1016/j.freeradbiomed.2012.10.530 PMC4711372

[9] EI-Seedi HR, EI-Said AM, Khalifa SA, Goransson U, Bohlin L, Borg-Karlson AK, et al (2012). Biosynthesis, natural sources, die-tary intake, pharmacokinetic properties, and biological activities of hydroxy-cinnamic acids. J Agric Food Chem, 60:10877-10895. 22931195 10.1021/jf301807g

[10] Rebelo MJ, Rego R, Ferreira M, Oliveira MC (2013). Comparativestudy of the antioxidant capacity and polyphenol content of Douro wines by chemical and electrochemical methods. Food Chem, 141:566-573. 23768395 10.1016/j.foodchem.2013.02.120

[11] David IG, Bizgan AM, Popa DE, Buleandra M, Moldovan Z, Badea IA, et al (2015). Rapid determination of total polyphenolic content in tea samples based on caffeic acid voltammetric behaviour on a disposable graphite electrode. Food Chem, 173:1059-1065. 25466125 10.1016/j.foodchem.2014.10.139

[12] U Rehman M, Sultana S (2011). Attenuation of oxidative stress, inflammation and early markers of tumor promotion by caffeic acid in Fe-NTA exposed kidneys of Wistar rats. Mol Cell Biochem, 357:115-124. 21647614 10.1007/s11010-011-0881-7

[13] Min J, Shen H, Xi W, Wang Q, Yin L, Zhang Y, et al (2018). Synergistic anticancer activity of combined use of caffeic acid with paclitaxel enhances apoptosis of non-small-cell lung cancer H1299 cells in vivo and in vitro. Cell Physiol Biochem, 48:1433-1442. 30064123 10.1159/000492253

[14] Liu Y, Qui S, Wang L, Zhang N, Shi Y, Zhou H, et al (2019). Reproductive and developmental toxicity study of caffeic acid in mice. Food Chem Toxicol, 123:106-112. 30366071 10.1016/j.fct.2018.10.040

[15] Kiel MJ, Yilmaz OH, Iwashita T, Yilmaz OH, Terhorst C, Morrison SJ (2005). SLAM family receptors distinguish hematopoietic stem and progenitor cells and reveal endothelial niches for stem cells. Cell, 121:1109-1121. 15989959 10.1016/j.cell.2005.05.026

[16] Kook SH, Yun CY, Sim HJ, Bhattarai G, Lee BC, Lee KY, et al (2016). Smad4 in osteoblasts exerts a differential impact on HSC fate depending on osteoblast maturation stage. Leukemia, 30:2039-2046. 27271228 10.1038/leu.2016.133

[17] Janzen V, Forkert R, Fleming HE, Saito Y, Waring MT, Dombkowski DM, et al (2006). Stem-cell ageing modified by the cyclin-dependent kinase inhibitor p16INK4a. Nature, 443:421-426. 16957735 10.1038/nature05159

[18] Akashi K, Traver D, Miyamoto T, Weissman IL (2000). A clonogenic common myeloid progenitor that gives rise to all myeloid lineages. Nature, 404:193-197. 10724173 10.1038/35004599

[19] Gülçin I (2006). Antioxidant activity of caffeic acid (3,4-dihydroxycinnamic acid). Toxicology, 217:213-220. 16243424 10.1016/j.tox.2005.09.011

[20] Liou GY, Storz P (2010). Reactive oxygen species in cancer. Free Radic Res, 44:479-496. 20370557 10.3109/10715761003667554 PMC3880197

[21] Devipriya N, Sudheer AR, Menon VP (2008). Caffeic acid protects human peripheral blood lymphocytes against gamma radiation-induced cellular damage. J Biochem Mol Toxicol, 22:175-186. 18561333 10.1002/jbt.20228

[22] Lukmanul Hakkim F, Miura M, Matsuda N, Alharassi AS, Guillemin G, Yamauchi M, et al (2014). An in vitro evidence for caffeic acid, rosmarinic acid and trans cinnamic acid as a skin protectant against γ-radiation. Int J Low Radiation, 9:305-316.

[23] Rajenda Prasad N, Karthikeyan A, Karthikeyan S, Reddy BV (2011). Inhibitory effect of caffeic acid on cancer cell proliferation by oxidative mechanism in human HT-1080 fibrosarcoma cell line. Mol Cell Biochem, 349:11-19. 21116690 10.1007/s11010-010-0655-7

[24] Chang WC, Hsieh CH, Hsiao MW, Lin WC, Hung YC, Ye JC (2010). Caffeic acid induces apoptosis in human cervical cancer cells through the mitochondrial pathway. Taiwan J Obstet Gynecol, 49:419-424. 21199742 10.1016/S1028-4559(10)60092-7

[25] Brautigan DL, Gielata M, Heo J, Kubicka R, Ilkins LR (2018). Selectiv toxicity of caffeic acid in hepatocellular carcinoma cells. Biochem Biophys Res Commun, 505:612-617. 30278886 10.1016/j.bbrc.2018.09.155

[26] Chen YJ, Liao HF, Tsai TH, Wang SY, Shiao MS (2005). Caffeic acid phenethyl ester preferentially sensitizes CT26 colorectal adenocarcinoma to ionizing radiation without affecting bone marrow radioresponse. Int J Radiat Oncol Biol Phys, 63:1252-1261. 16253780 10.1016/j.ijrobp.2005.08.001

[27] Schlüter K, Gassmann P, Enns A, Korb T, Hemping-Bovenkerk A, Hölzen J, et al (2006). Organ-specific metastatic tumor cell adhesion and extravasation of colon carcinoma cells with different metastatic potential. Am J Pathol, 169:1064-1073. 16936278 10.2353/ajpath.2006.050566 PMC1698818

[28] Clamp A, Danson S, Nguyen H, Cole D, Clemons M (2004). Assessment of therapeutic response in patients with metastatic bone disease. Lancet Oncol, 5: 607-616. 15465464 10.1016/S1470-2045(04)01596-7

[29] Roth ES, Fetzer DT, Barron BJ, Joseph UA, Gayed IW, Wan DQ (2009). Does colon cancer ever metastasize to bone first? a temporal analysis of colorectal cancer progression. BMC Cancer, 274:1-6. 10.1186/1471-2407-9-274 PMC2734866 19664211

